# P062. Sensory modalities during dreams in migraine

**DOI:** 10.1186/1129-2377-16-S1-A63

**Published:** 2015-09-28

**Authors:** Luca Giani, Carlo Lovati, Roberta Casazza, Claudio Mariani

**Affiliations:** UO Neurologia, AO L. Sacco, Università degli Studi di Milano, Milan, Italy

## Background

Migraine is a primary headache characterized by recurrent episodes of unilateral head pain, associated to vegetative symptoms (nausea) and alterations in sensory experiences: photophobia and phonophobia are diagnostic criteria, osmophobia, although not diagnostic, seems to be very specific[1]. Dream is a universal mental activity present during sleep, characterized by hallucinatory production. Subjects may recall experiences in different sensory modalities, such as visual, auditory, olfactory and gustatory sensations[2]. In a previous study, through an anamnestic questionnaire, we found that migraine patients were more prone than non-migraineurs to recall gustatory and olfactory dreams[3]. We designed a study aiming to eliminate the possible bias due to the retrospective analysis.

## Materials and methods

We enrolled 104 subjects (69 F, 35 M; mean age 35.39±14.10; range 20-76), of which 59 controls not suffering from any kind of primary headache (30 F; mean age 32.75±13.29; range 20-76), 17 migraine with aura (MA, 17 F; mean age 40.53±12.27; range 24-59), 28 migraine without aura (MO, 22 F; mean age 37.86±15.87; range 23-70). Headache diagnosis was made according to the ICHD-III beta criteria. All patients filled in an ad hoc questionnaire each morning upon awakening for 30 consecutive days. The questionnaire asked the patients if they remembered dreaming in the past night, and investigated the presence of different sensory modalities (i.e., presence of color, black and white, acoustic, olfactory/gustatory sensations). Olfactory and gustatory experiences were included in the same question, as subjects of a previous study reported difficulty in discriminating between these two sensory modalities. We examined the prevalence of subjects reporting each sensory experience at least once in 30 days, and compared different diagnostic groups using Chi square test or Fisher exact test when appropriate.

## Results

**Figure 1:**
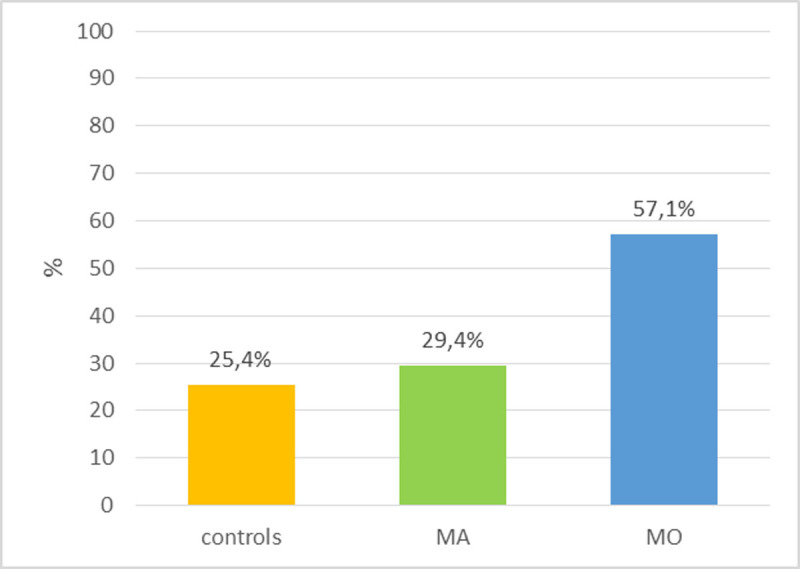
**Proportion of subjects reporting olfactory/gustatory sensations**. Legend: MA, migraine with aura; MO, migraine without aura.

There was no differences between migraineurs and controls about the presence of color (93.3% vs 94.9%), black and white (37.8% vs 33.9%), and auditory (97.8% vs 86.4%) experiences. More migraineurs than controls reported dreaming with olfactory/gustatory (46.7% vs 25.4%, p 0.037 at Fisher exact test) sensations at least once. More MO patients reported olfactory/gustatory sensations with respect to MA patients and controls (respectively 57.1% vs 29.4% vs 25.4%, p 0.013) (figure 1).

## Conclusions

With this study, even eliminating the possible bias of retrospective analysis, we confirmed that migraine subjects experience gustatory and olfactory sensations during dreams more frequently than subjects without migraine. These results suggest a peculiar functioning of brain structures such as the amygdala and the hypothalamus in the migraine brain.

Written informed consent to publication was obtained from the patient(s).
